# Measurement of cardiac output using the transpulmonary thermodilution method in the presence of high extravascular lung water in a pediatric animal model

**DOI:** 10.1186/cc9467

**Published:** 2011-03-11

**Authors:** A Nusmeier, S Vrancken, JG Van der Hoeven, J Lemson

**Affiliations:** 1 Radboud University Nijmegen Medical Centre, Nijmegen, the Netherlands

## Introduction

Cardiac output (CO) can be measured using the transpulmonary thermodilution (TPTD) technique. TPTD is considered to be the gold standard in pediatric patients. We studied the influence of high levels of EVLW on the reliability of CO measurement using the TPTD technique in a pediatric animal model.

## Methods

Anesthetized, mechanically ventilated lambs were instrumented with a COLD^® ^(Pulsion Medical Systems, Munich, Germany) catheter and underwent repetitive saline lavage (10 to 30 ml/kg) of the lung. CO was measured using the single indicator TPTD method (COTPTD) and compared with simultaneous measurement of CO using an ultrasound perivascular flowprobe (Transonic Systems, USA) around the main pulmonary artery (COMPA). EVLW was assessed by the transpulmonary double indicator technique with intravenous injections of ice-cold indocyanine green (ICG).

## Results

**Figure 1:**
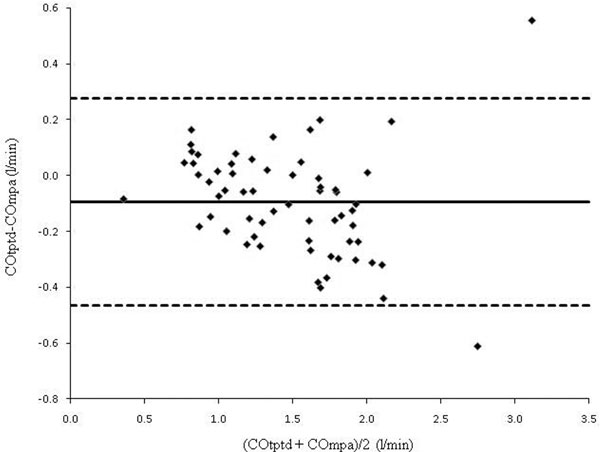
**Bland-Altman analysis of COtptd and COmpa**.

A total of 62 simultaneous measurements in 11 lambs were analyzed. The mean body weight was 8.6 (range 4.1 to 12.3) kg. The initial EVLWI was 13.8 (range 9.3 to 21.5) ml/kg. After lung injury this increased to 38.3 (range 16.2 to 60.9) ml/kg. The mean COMPA was 1.52 (range 0.40 to 3.05) l/minute. The correlation coefficient between the COMPA and COTPTD was 0.93. The Bland-Altman analysis showed a mean bias of -0.09 l/minute (limits of agreement ± 0.37 l/minute) (Figure 1). The percentage error was 25%.

## Conclusions

In circumstances of largely increased extravascular lung water, CO can reliably be measured using the TPTD technique.

